# *Aliivibrio wodanis* as a production host: development of genetic tools for expression of cold-active enzymes

**DOI:** 10.1186/s12934-019-1247-1

**Published:** 2019-11-11

**Authors:** Jenny Johansson Söderberg, Miriam Grgic, Erik Hjerde, Peik Haugen

**Affiliations:** Department of Chemistry and Center for Bioinformatics (SfB) and The Norwegian Structural Biology Centre (NorStruct), Faculty of Science and Technology, UiT -The Arctic University of Norway, 9037 Tromsø, Norway

**Keywords:** *Aliivibrio wodanis*, Heterologous protein production, Expression host, Psychrophilic enzymes, Cold adaptation, Marine biotechnology

## Abstract

**Background:**

Heterologous production of cold-adapted proteins currently represents one of the greatest bottlenecks in the ongoing bioprospecting efforts to find new enzymes from low-temperature environments, such as, the polar oceans that represent essentially untapped resources in this respect. In mesophilic expression hosts such as *Escherichia coli*, cold-adapted enzymes often form inactive aggregates. Therefore it is necessary to develop new low-temperature expression systems, including identification of new host organisms and complementary genetic tools. Psychrophilic bacteria, including *Pseudoalteromonas haloplanktis*, *Shewanella* and *Rhodococcus erythropolis* have all been explored as candidates for such applications. However to date none of these have found widespread use as efficient expression systems, or are commercially available. In the present work we explored the use of the sub-Arctic bacterium *Aliivibrio wodanis* as a potential host for heterologous expression of cold-active enzymes.

**Results:**

We tested 12 bacterial strains, as well as available vectors, promoters and reporter systems. We used RNA-sequencing to determine the most highly expressed genes and their intrinsic promoters in *A. wodanis*. In addition we examined a novel 5′-fusion to stimulate protein production and solubility. Finally we tested production of a set of “difficult-to-produce” enzymes originating from various bacteria and one Archaea. Our results show that cold-adapted enzymes can be produced in soluble and active form, even in cases when protein production failed in *E. coli* due to the formation of inclusion bodies. Moreover, we identified a 60-bp/20-aa fragment from the 5′-end of the AW0309160_00174 gene that stimulates expression of Green Fluorescent Protein and improves production of cold-active enzymes when used as a 5′-fusion. A 25-aa peptide from the same protein enhanced secretion of a 25-aa-sfGFP fusion.

**Conclusions:**

Our results indicate the use of *A. wodanis* and associated genetic tools for low-temperature protein production and indicate that *A. wodanis* represents an interesting platform for further development of a protein production system that can promote further cold-enzyme discoveries.

## Background

The first recombinant proteins were produced in *Escherichia coli* in 1976 [1]. Since then the production of recombinant proteins in *E. coli* has had great impact on our lives, for example, by eliminating the need to extract proteins from large volumes of native biological material [2]. However, although the production of recombinant proteins is a well-developed method, there are still challenges to overcome, and many variables to consider, such as, the expression host, plasmid vectors, promoters, selection markers, affinity tags, and fusion partners. Strategies to increase production and avoid problems have been comprehensively reviewed in the past [3–6]. In particular, psychrophilic (cold-adapted) enzymes are difficult to express in conventional mesophilic hosts (such as *E. coli*) [7]. Psychrophilic enzymes originate from organisms living in cold environments, and with 80% of the earth’s biosphere being below 5 °C [8], the organisms living in these environments represent a largely untapped resource with respect to enzyme discovery.

Cold adapted enzymes are generally characterized by a higher catalytic activities at low temperatures, compared to their mesophilic homologs with rate enhancements up to tenfold [9]. Current understanding of this phenomenon are that the whole protein, or parts of it such as the active site, are destabilized due to the weakening of inter- and intramolecular bonds, thus increasing flexibility at low temperatures [10, 11]. Consequently, cold-adapted enzymes are thermolabile, melting at relatively low temperatures meaning a relatively small increase in temperature can lead to inactive protein. These properties can be highly beneficial in some commercial and biotechnological applications [10, 12, 13]. One example is the use of cold-active enzymes as components of detergents, which reduces the need to heat water during washing. Another example is shrimp alkaline phosphatase which is completely inactivated after 5 min at 65 °C compared to calf intestinal alkaline phosphatase where a typical protocol for > 95% inactivation is 10 min at 70 °C. Less aggressive heat inactivation can improve the quality of the final sample and simplify experimental protocols. Cold-active enzymes are therefore replacing some of the mesophilic enzymes already on the market. Progress in development of new cold-active enzymes is slow and severely hampered by the lack of efficient protocols for the production of proteins in active form. There is an urgent need to develop new tools, methods and expression hosts for low-temperature protein production.

Different strategies to improve expression of challenging enzymes in *E. coli* have been tried. These include decreasing the temperature during fermentation and manipulating the cell’s folding machinery by co-expressing chaperones/chaperonins/foldases, with a well-known example being the *E. coli* ArcticExpress strain [14, 15]. Another approach has been to refold proteins post expression, using (e.g., Urea, *N*-lauroylsarcosine, Dithiothreitol or 2-mercaptoethanol) and then refolding to active protein by removing the denaturant. However, for many proteins (especially enzymes), conventional refolding methods are time consuming with recovered yields of active proteins being low due to use of aggressive chemicals during protein refolding [16–18]. A promising approach to improving expression of cold-active proteins is to use bacterial isolates that are naturally adapted to very low temperature as expression hosts. In microorganisms that live in very cold locations, such as the bacterium *Sphingopyxis alaskensis* [19], a range of cellular processes are involved to ensure survival during exposure to low-temperature.

There have been several attempts in utilizing psychrophilic bacteria as potential expression hosts. Yu et al. [20] used the psychrophilic strain *Pseudoalteromonas* sp. SM20429 to successfully produce three cold-adapted *Pseudoalteromonas* enzymes. These proteins, protease (pseudoalterin), UDP-GlcNac 2-epimerase and UDP-ManNAc dehydrogenase) were affinity purified in active form [20]. In addition, development of an expression system based on *Pseudoalteromonas haloplanktis* TAC125 including successful protein production at sub-zero temperatures [21–29]. Despite successful use of *Pseudoalteromonas* strains for production of cold-adapted proteins, one of the remaining issues is demonstration of protein expression from a wider phylogenetic range. Miyake et al. addressed this using *Shewanella* sp. strain Ac10 and broad-host-range vector (pJRD215) to express β-lactamase, three putative peptidases (PepF, LAP, and PepQ), and a putative glucosidase (BglA), all originating from the psychrophilic bacterium, *Desulfotalea psychrophila* DSM12343 [30]. However, despite these successes, to date there are still no broadly available cold adapted protein production systems. The work described above is encouraging, but also shows that development of efficient systems for cold-temperature expression is far from trivial. Even though some of the criteria for successful expression have been met there are still challenges to overcome. For example, even though there are reports of successful and stable growth of psychrophilic bacteria and protein production in fermenters under industrial-like conditions to very high densities, more fine-tuning and optimization of protein production it still required [25, 28, 29]. Perhaps the most important prerequisites for a successful system are that the expression host must be able to grow fast to high densities at low temperatures and that genetic tools to introduce and genetically modify the bacterial genomic DNA are available. Other essential criteria are that the expression host can grow in cheap growth media without requiring expensive supplements, which is especially important for industrial applications. Finally, the bacterium should not be pathogenic. One major drawback with developing new expression systems is that it requires extensive efforts to develop molecular tools, methods and protocols in order to achieve efficient production of target proteins.

Within our bacterial strain collection, mostly comprising of Sub-Arctic marine isolates, we noted that several *Aliivibrio wodanis* strains, of the *Vibrionaceae* family, grew fast at low temperatures. This observation and the fact that we had established genetic tools for closely related species e.g. *Aliivibrio salmonicida* [31–33] encouraged us to pursue *A. wodanis* as a potential expression host for cold-adapted enzymes. In this work, we have evaluated properties of 12 *A. wodanis* strains, including growth, antibiotic resistance, the ability to take up DNA, to integrate DNA into its chromosomes, and to produce reporter systems [Green fluorescent protein (GFP), and Red Fluorescent Protein (RFP)] [34]. Promising strain candidates were further tested for production of cold-adapted enzymes originating from various genetic sources. Earlier attempts to produce some of these enzymes in *E. coli* had failed to generate soluble proteins. In order to increase expression levels in *A. wodanis*, we used RNA-sequencing to identify highly expressed genes and to design a 60-nt/20-aa fusion to increase expression levels. Overall, the results presented here show that *A. wodanis* has the capacity to produce cold-adapted and “difficult-to-produce” proteins in soluble and active form, and in this respect outperforms *E. coli*.

## Results and discussion

### Selection of strains and genetic tools

**Fig. 1:**
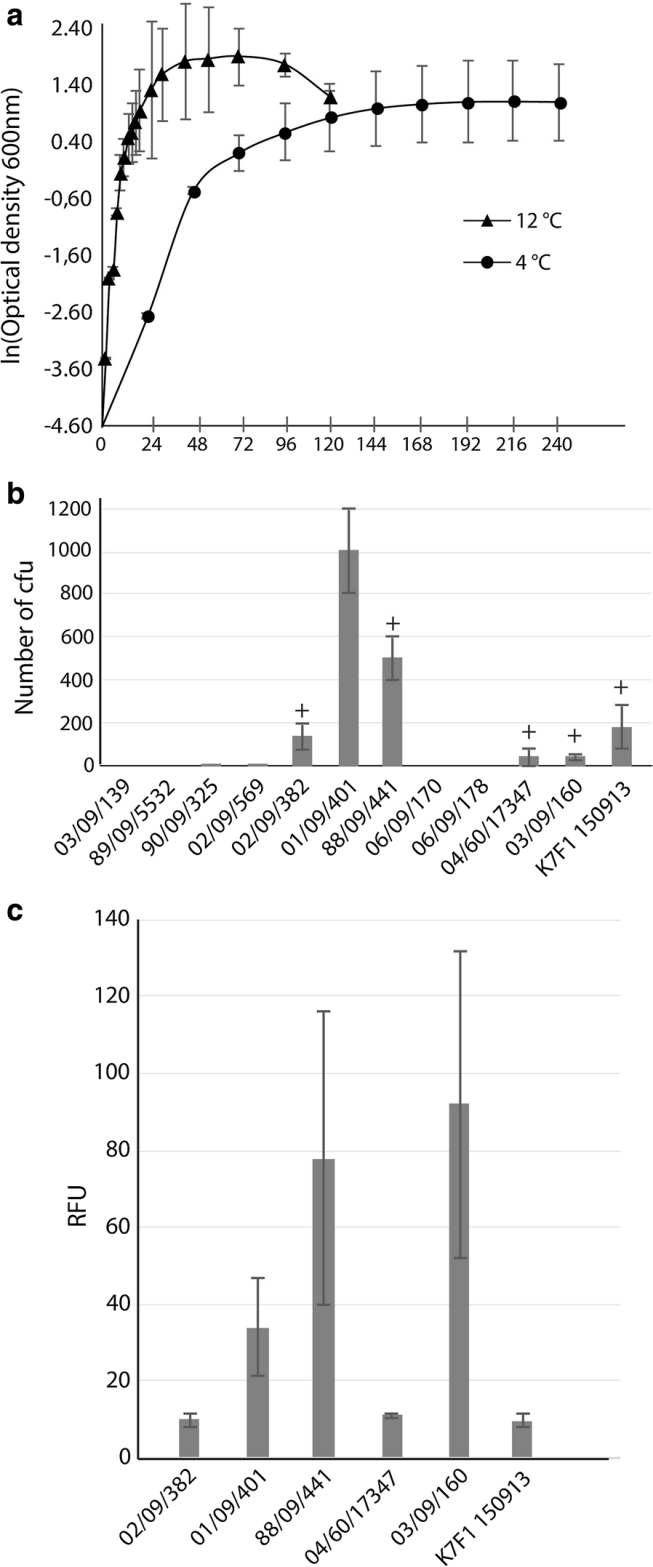
Selection of *A. wodanis* strains. **a** Growth curve for *A. wodanis* strain 03/09/160 at 4 and 12 °C. Similar growth pattern was observed for all thirteen tested strains. Error bars represent standard deviation between three replicates. **b** Conjugation efficiency for 12 *A. wodanis* strains. Bars show uptake of pTM214 vector. Plus signs (+) indicate integration of pNQ705 vector into chromosome, strains that could not integrate pNQ705 vector into chromosome are missing (+) sign above bars. **c** Expression of RFP in six *A. wodanis* strains, that are able to uptake vectors via conjugation, containing pVSV208. Bars indicate measured relative fluorescence. *RFU* relative fluorescence units

Table 1 shows the 12 *A. wodanis* strains that were selected from our in-house strain collection to identify promising expression host candidates. Strains were tested for growth rate, resistance against some commonly used antibiotics (in biotechnological applications), and conjugation efficiency (uptake and stability of plasmids). Preliminary tests showed that *A. wodanis* does not grow well at temperatures above 20 °C and has an optimal growth rate in the range 12–18 °C. All of the strains tested showed very similar growth profiles. Figure 1a shows growth in standard culture flasks for one representative strain (03/09/160). *A. wodanis* grows considerably faster at 12 °C than at 4 °C with doubling times of 2.5 and 25 h, respectively. Moreover, *A. wodanis* uses 72 and 144 h to reach maximum optical densities, at 12 °C and 4 °C, respectively. At 12 °C the bacterium reaches OD_600nm_ = 7 in standard LB medium supplemented with 2.5% NaCl.

**Table 1:** Properties associated with *A. wodanis* strains used in this study

Strain	Origin	GenBank^a^	Antibiotics resistance						Refs.
			CHL 2 µg/mL	TET 10 µg/mL	CAR 100 µg/mL	CAR 200 µg/mL	KAN 50 µg/mL	KAN 100 µg/mL	
06/09/139	*S. salar*	GCA_000953695.1	NA	NA	NA	NA	NA	NA	[54]
89/09/5532 (1)	*S. salar*	JQ361718	0	0	1	1	1	0.5	[55]
90/09/325 (5)	*S. salar*	JQ361719	0	0	1	1	1	0.5	[55]
02/09/569 (7)	*S. salar*	JQ361720	0	0	1	1	1	0.5	[55]
02/09/382 (8)	*S. salar*	JQ361721	0	0	1	1	1	0.5	[55]
01/09/401 (11)	*S. salar*	JQ361722	0	0	1	1	1	0.5	[55]
88/09/441 (12) (T)	*S. salar*	JQ361723	0	0	0.5	0.5	1	0.5	[55]
06/09/170 (27)	*S. salar*	JQ361727	0	0	1	1	0.5	0.5	[55]
06/09/178 (29)	*O.mykiss*	JQ361731	0	0	1	1	1	0.5	[55]
04/60/17347 (35)	*G.morhua*	JQ361730	0	0	1	1	1	0.5	[55]
03/09/160 (37)	*S. salar*	JQ361729	0	0	1	1	1	0.5	[55]
K7F1 150913	*S. salar*	–	0	0	1	1	1	0.5	This study

*S. salar *: *Salmo salar* (Atlantic salmon); *O. mykiss *: *Oncorhynchus mykiss* (Rainbow trout); *G. morhua *: *Gadus morhua* (Atlantic cod); CHL: chloramphenicol; TET: Tetracycline; CAR: Carbenicillin; KAN: Kanamycin; 0: Susceptible; 0.5: Intermediate Susceptible; 1: Resistant. Abbreviations for antibiotics follow that of the *American Society for Microbiology*; NA: not analysed

^a^GenBank accession numbers. The accession number for strain 06/09/139 refers to the genome assembly

We then tested *A. wodanis* for resistance to antibiotics, four of which are commonly used in biotechnological applications, namely carbenicillin, kanamycin, tetracycline and chloramphenicol. Resistance of *A. wodanis* strains to ampicillin, nitrofurantoin, tetracyclines, cefoxitin and sulfamethoxazole has been reported [35]. The 12 strains used in this study were sensitive to chlorampenichol and tetracycline, had intermediate sensitivity to kanamycin and showed resistance to carbenicillin **(**see Table 1).

To test the ability of *A. wodanis* to take up plasmids, each strain was conjugated with the replicative vector pTM214 [36]. In *E. coli,* the mCherry fluorescent protein is constitutively expressed from pTM214, whereas expression of mCherry in *A. wodanis* requires addition of IPTG to the growth medium. The inability of *E. coli* to grow under propagation conditions permissive for *A. wodanis* provides a convenient method to select for *A. wodanis* cells after conjugation. As, only *A. wodanis* colonies carrying the pTM214 plasmid grow well to visible size. In addition, as the growth medium (agar plates) did not contain IPTG, *A. wodanis* colonies are identifiable by their lack of mCherry expression which is constitutively expressed from pTM214 in *E. coli*. Figure 1b shows that six of the 12 *A. wodanis* strains (i.e., strains 02/09/382, 01/09/401, 88/09/441, 04/60/17347, 03/09/160 and K7F1 150913) readily receive and accept the foreign plasmid (pTM214) through conjugation. The six recipient strains were then tested for their ability to receive and integrate the integrative plasmid pNQ705 [37]. A 250 bp DNA region homologous to the *A. wodanis ainS* gene was first inserted into pNQ705 to promote genomic integration. The test was performed using three experimental replicates for each strain and regarded as positive if at least one integration into the bacterial host genome was found. Figure 1b shows that integration was found in strains 02/09/382, 88/09/441, 04/60/17347, 03/09/160 and K7F1 150913, i.e. five of six tested strains.

Next, we used the plasmid pVSV208 to determine the capacity of strains to produce a reporter protein, the red fluorescent protein (RFP), under antibiotic (chloramphenicol) pressure. Only strains that received the pTM214 plasmid were tested (see above). First, production was monitored by examining the morphology (color) of colonies using a fluorescent microscope. Bright red colonies of strains 01/09/401 and 03/09/160 indicated strong RFP expression. Colonies of strains 02/09/382, 04/60/17347 and K7F1 150913 were less bright. We observed considerable variation in strain 88/09/441 where colonies ranged from bright red to white color indicating uneven expression levels in individual colonies. Further, RFP production was monitored in liquid cultures by measuring fluorescence (588 nm) in the supernatant of lysed cell cultures. Figure 1c shows that the relative fluorescence values are in good agreement with the colony morphologies described above. Again, strains 01/09/401, 88/09/441 and 03/09/160 produced highest fluorescence intensities.

In summary, based on the results described above strains 01/09/401 and 03/09/160 are the most promising candidates for production of proteins. Both strains grow well at low temperature to relatively high optical densities. Both can receive plasmids via conjugation, and can stably produce RFP. Strain 03/09/160 is additionally capable of integrating plasmids efficiently into its genome.

### Production of green fluorescent protein reporter system at low temperature

**Fig. 2:**
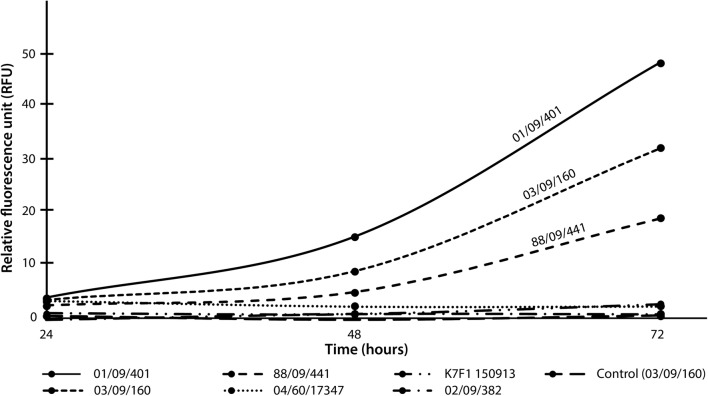
Expression of GFP in *A. wodanis*. Expression of GFP (green fluorescent protein) was compared in six *A. wodanis* strains in a time series over 72 h from the plasmid pTM214_HIS-GFP. Expression was induced by adding 0.1 mM IPTG from the start of the experiment. Highest relative fluorescent unit (RFU) values were recorded from strains 01/09/401, 03/09/160 and 88/09/441. Strain 03/09/160 with no plasmid was used as control

Next, we tested the ability of *A. wodanis* strains to support production of a His-tagged green fluorescent protein (GFP). In a pre-experiment, cultures of *A. wodanis* 03/09/160 containing pTM214_His-GFP were IPTG induced and showed that expression is strongly elevated from the *P*_*trc*_ promoter after 48 h and 72 h (Additional file 1: Fig. S1). Figure 2 shows the result of a time series experiment with GFP production in six *A. wodanis* strains (02/09/382, 01/09/401, 88/09/441, 04/60/17347, 03/09/160 and K7F1 150913). Strains 01/09/401 and 03/09/160, followed by strain 88/09/441 produce highest RFU values, which is in overall agreement with the RFP expression data (above), although the exact order is not the same.

### Protein production, purification and activity of cold-adapted enzymes from the *Aliivibrio* genus

**Fig. 3:**
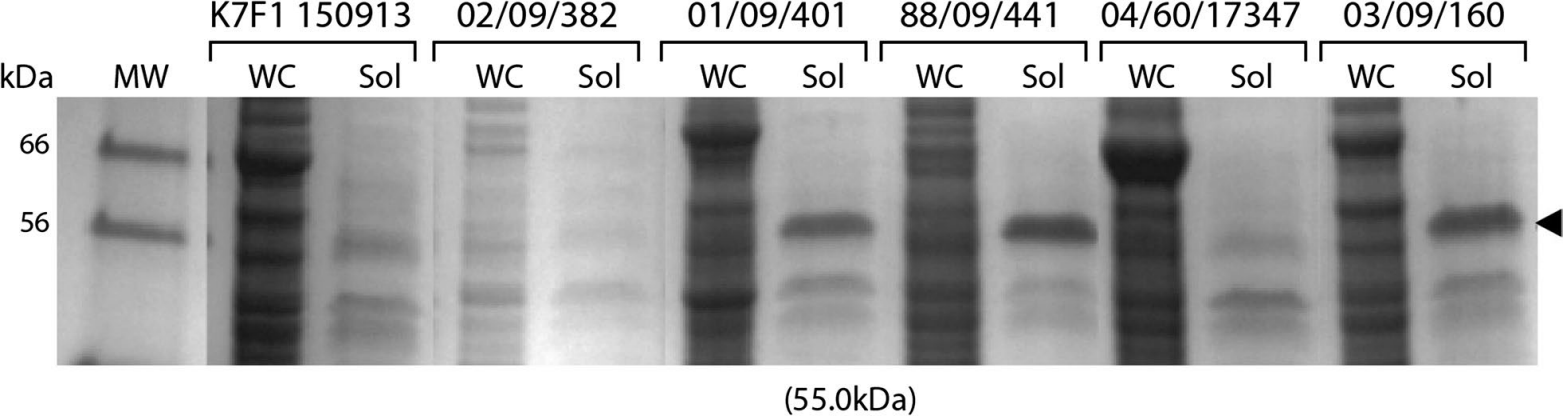
Expression of Exonuclease I from *A. salmonicida* (AsExoI). Expression of AsExoI was compared in six *A. wodanis* strains. Arrowhead denotes bands with molecular weight (55.0 kDa) corresponding to AsExoI (verified by mass spectrometry). Produced protein was purified using His-tagged magnetic beads. *WC* whole cell, *Sol* IMAC-purified protein fraction, *MW* molecular weight marker

Having shown production of three reporter systems, mCherry, RFP and GFP, we next wanted to test “difficult-to-produce” cold-adapted enzymes. Test-cases were selected projects at the Norwegian Structure Biology Center (NorStruct) that had been terminated due to unsuccessful or poor expression in *E. coli*. As test-cases we first selected two enzymes, Exonuclease I (AsExoI) and DNA Polymerase II (AsPolII) both from *Aliivibrio salmonicida.* The rationale was that expression had previously failed in *E. coli* due to the formation of inclusion bodies and that the proteins originate from a close relative of *A. wodanis*, which was likely to improve the chances of successful expression. To determine which of the six tested strains was preferable for test production, each was transformed with pTM214 vector containing the AsExoI gene under IPTG inducible promoter (*P*_*trc*_). Figure 3 shows AsExoI protein bands visible for strains 01/09/401, 88/09/441 and 03/09/160, which is in agreement with RFP and GFP expression. Note that soluble (Sol) samples in Fig. 3 represent affinity purified sample from the His-tag on recombinant AsExoI. Based on this result and the additional characterizations described above, strain 03/09/160 was chosen as the “standard” expression strain for the remaining part of this study.

**Fig. 4:**
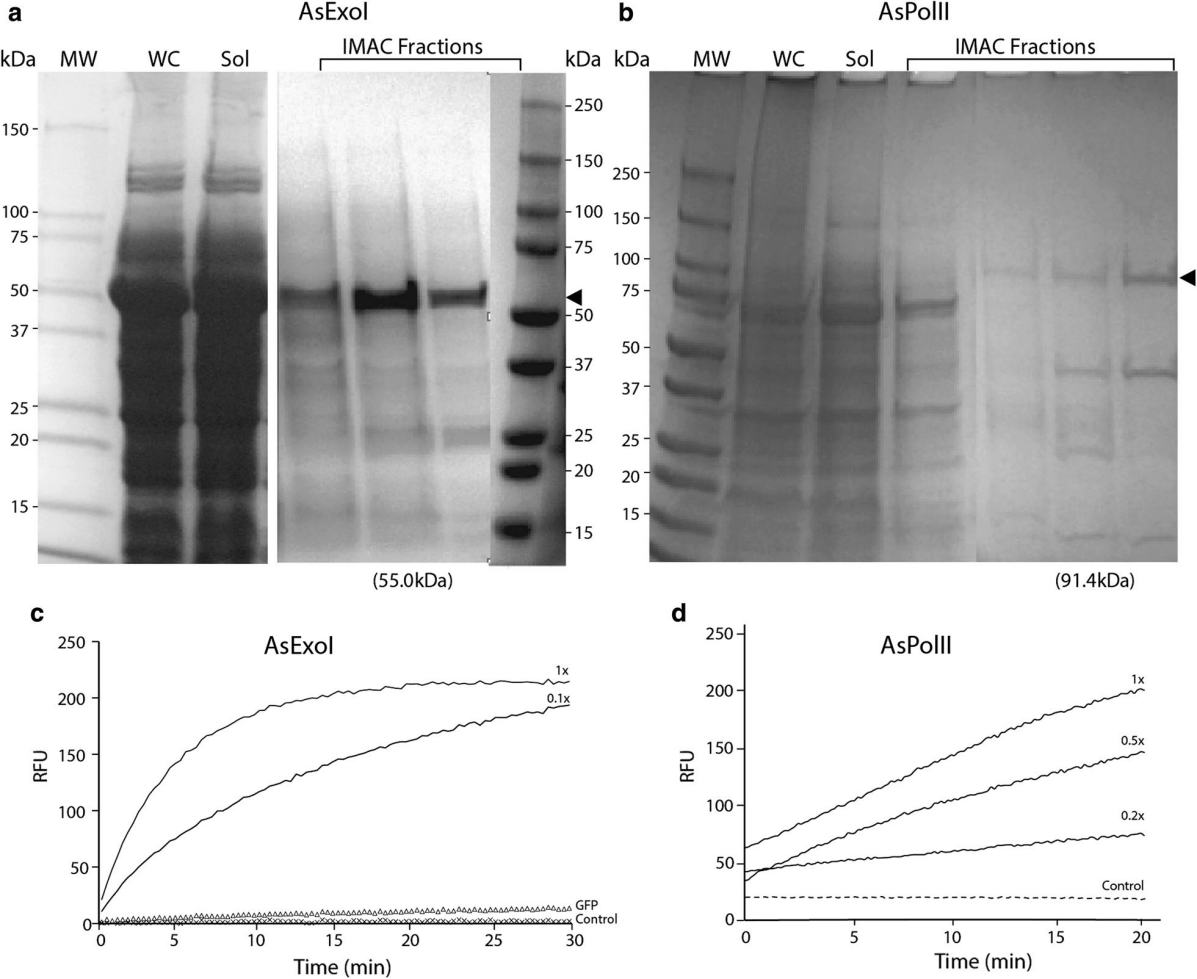
Expression, purification and activity of AsExoI and AsPolII. Coomassie-stained SDS acrylamide gels showing expressed and affinity purified 6 × His-AsExoI (**a**) and 6 × His-AsPolII (**b**). MW = molecular weight marker (Biorad protein standard), WC = whole cell extract, Sol = lysate soluble protein fraction. Immobilized metal affinity chromatography (IMAC) was done to purify 6 × His-tagged proteins, and proteins eluted in IMAC fractions are shown on the gels. Molecular weights of AsExoI ans AsPolII are theoretically 55.0 kDa and 91.4 kDa. Arrows heads indicate bands on geld that were identified as the desired enzyme targets. The activity of AsExoI (**c**) and AsPolII (**d**) was monitored by adding increasing concentrations of enzyme to molecular beacon substrate

Figure 4 shows production, purification and activity of enzymes AsExoI and AsPolII. They were both expressed using strain 03/09/160, affinity purified on an IMAC column and visualized by SDS-PAGE. Bands representing both proteins are clearly visible and specific fluorescence molecular beacon-based assays showed dose-dependent exonuclease and DNA polymerase activities, respectively (see “Methods” for assay details), thus demonstrating that the proteins are expressed in active form at low temperature. To test for endogenous (background) exonuclease activity from the *A. wodanis* host, we also expressed GFP in a separate A. wodanis culture as a control. No residual exonuclease activity was detected in the control (Fig. 4c), which support that only activity of recombinant AsExoI exonuclease was measured in our assay.

Finally, we compared expression of the cold-active enzyme AsExoI in (*i*) *A. wodanis* 03/09/160 at 12 °C for 3 days, and (ii) in *E. coli* at 20 °C for 5 h (see Additional file 1: Fig. S2). The result shows that AsExoI is expressed in high quantities in *E. coli*, but is lost from the soluble protein fraction. In contrast, AsExoI is expressed in lower quantities in *A. wodanis*, but is readily affinity purified from the soluble protein fraction and produce a distinct band on the gel.

### A 60 bp/20-aa fragment originating from a highly expressed gene (AW0309160_00174) elevates expression of a gfp fusion

In an attempt to increase the protein production in *A. wodanis* we adapted a strategy, in which the 5′-end of a highly expressed genes is used as a fusion partner, and added to the 5′-coding region of the target gene. Examples include the use of the secretion signals from PelB OmpC and CelB [38–40]. Another strategy was that the addition of a strong RNA stem-loop to the mRNA 5′-end that could enhance expression [41]. To find the most highly expressed genes, we cultivated *A. wodanis* 03/09/160 under our standard growth conditions, harvested cells at OD_600nm_ = 2 (exponential phase) and used RNA-sequencing.

**Fig. 5:**
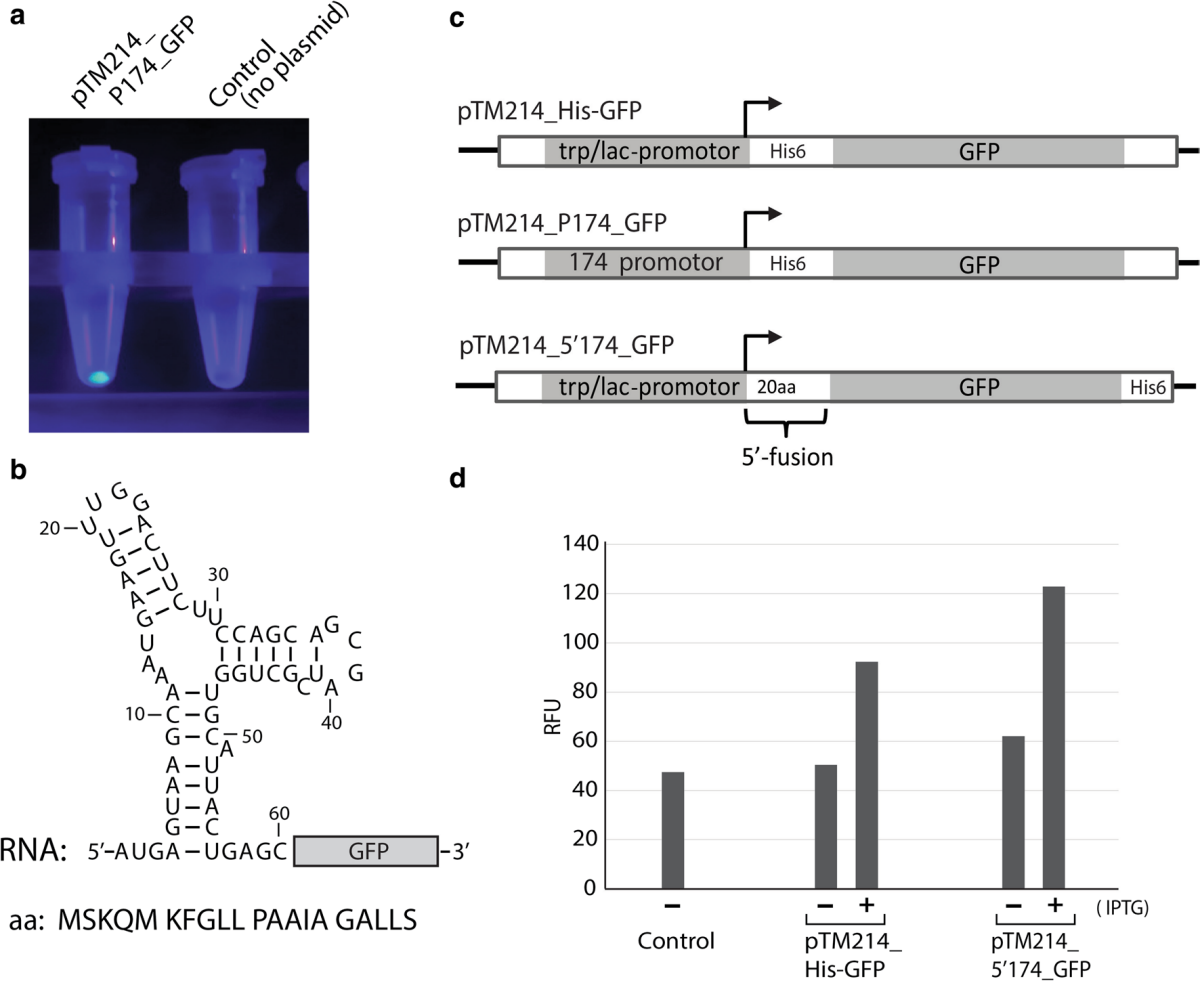
Expression of *gfp* using a 5′-fusion sequence from AW0309160_00174. AW0309160_00174 was by RNA-sequencing identified in this study as the most highly expressed gene under our standard growth conditions. **a** Picture shows cell pellets of *A. wodanis* containing pTM214_P174_GFP (contains 300-bp of the AW0309160_00174 gene promoter), or no plasmid (control) in microcentrifuge tubes subjected to UV light. Bright green color shows strong expression of *gfp.* **b** Secondary structure model of the first 60-nt of AW0309160_00174 mRNA. The sequence was used as a 5′-fusion to improve protein expression (*gfp* shown as example). **c** Schematic figure showing the expression cassettes of plasmids pTM214_His-GFP, pTM214_P174_GFP and pTM214_5′174_GFP. pTM214_P174_GFP contains a 300-bp region of the AW0309160_00174 promoter placed in front of *gfp,* and the latter contains a *P*_*trc*_ promoter in front of a 60-nt/20-aa 5′-fusion from AW0309160_00174 followed by *gfp*. pTM214_5′174_GFP was used as backbone for cloning and expression of non-*Aliivibrio* enzyme test cases. **c**, **d** Fluorescence measurements of *A. wodanis* containing no plasmid (Control), pTM214_His-GFP or pTM214_5′174_GFP. Samples with (+) or without (−) IPTG are shown. Values are expressed as relative fluorescence units (RFU)

Table 2 shows a list of the top ten most highly expressed genes in *A. wodanis* 03/09/160. Interestingly, the level of expression of gene AW0309160_00174 is 2.2 × higher than that of the second most highly expressed gene, Awod_I1528 and 3.1 × higher than that of number three on the list, Awod_I1596. A 300-bp region upstream of AW0309160_00174, the promoter region, was cloned into the pTM214 vector in front of *gfp* (the plasmid was named pTM214_P174_GFP) to verify that the promoter can support strong expression in *A. wodanis* (Fig. 5a).

**Table 2:** Top-ten most highly expressed genes in *A. wodanis* 03/09/160

Gene nr.	Product	Base mean
AW0309160_00174	Unknown	212,918.07
AW0309160_02815	Phosphoenolpyruvate carboxykinase [ATP]—phosphoenolpyruvate carboxylase (*pckA*)	97,425.05
AW0309160_02905	ATP synthase subunit beta—ATP synthase F1sector subunit beta—F-ATPase subunit beta (*atpD*)	69,249.87
AW0309160_02075	30S ribosomal protein S1 (*rpsA*)	51,427.88
AW0309160_02664	50S ribosomal protein L16 (*rplP*)	45,942.99
AW0309160_02634	Hypotetical protein	42,958.71
AW0309160_02907	ATP synthase subunit alpha—ATP synthase F1sector subunit alpha—F-ATPase subunit alpha (*atpA*)	40,648.19
AW0309160_02646	DNA-directed RNA polymerase subunit alpha—RNApolymerase subunit alpha—transcriptase subunit alpha (*rpoA*)	37,931.23
AW0309160_00936	Alanine dehydrogenase (*ald*)	33,312.94
AW0309160_02673	Elongation factor Tu (*tufB_1*)	30,288.95

Further analysis of AW0309160_00174 revealed that the first 60-bp of the 5′-coding region can potentially form a strong RNA secondary structure consisting of three base-paired regions, and two terminal loops (Fig. 5b). This 60-bp/20-aa sequence was next cloned into pTM214 in front of a *gfp*/C-terminal His-tag to monitor any stimulating effect on protein production (from *P*_*lac*_). This construct was named pTM214_5′174GFP-His (Fig. 5c). Figure 5d shows a comparison of *A. wodanis* cells expressing GFP from pTM214_His-GFP and pTM214_5′174GFP-His. The addition of the 5′-fusion (60-bp/20-aa) results in a moderate increase in fluorescence in both uninduced and induced cells.

### Production of cold-adapted enzymes from non-*Aliivibrio* microbial sources

The following four non-*Aliivibrio* enzymes were then selected for test-expression: (i) Exonuclease I (MvExoI) from *Moritella viscosa* (Gammaproteobacteria; Alteromonadales; Moritellaceae), (ii) ligase 1 (CsLig1) from *Cenarchaeum symbiosum* (Archaea; TACK group; Thaumarchaeota; Cenarchaeales; Cenarchaeaceae), (iii) ligase 6 (CpLig6) from *Colwellia psychrerythraea* (Gammaproteobacteria; Alteromonadales; Colwelliaceae) and (iv) alcohol dehydrogenase (AdhStrep) from *Streptomyces* (Actinobacteria; Streptomycetales; Streptomycetaceae).

**Fig. 6:**
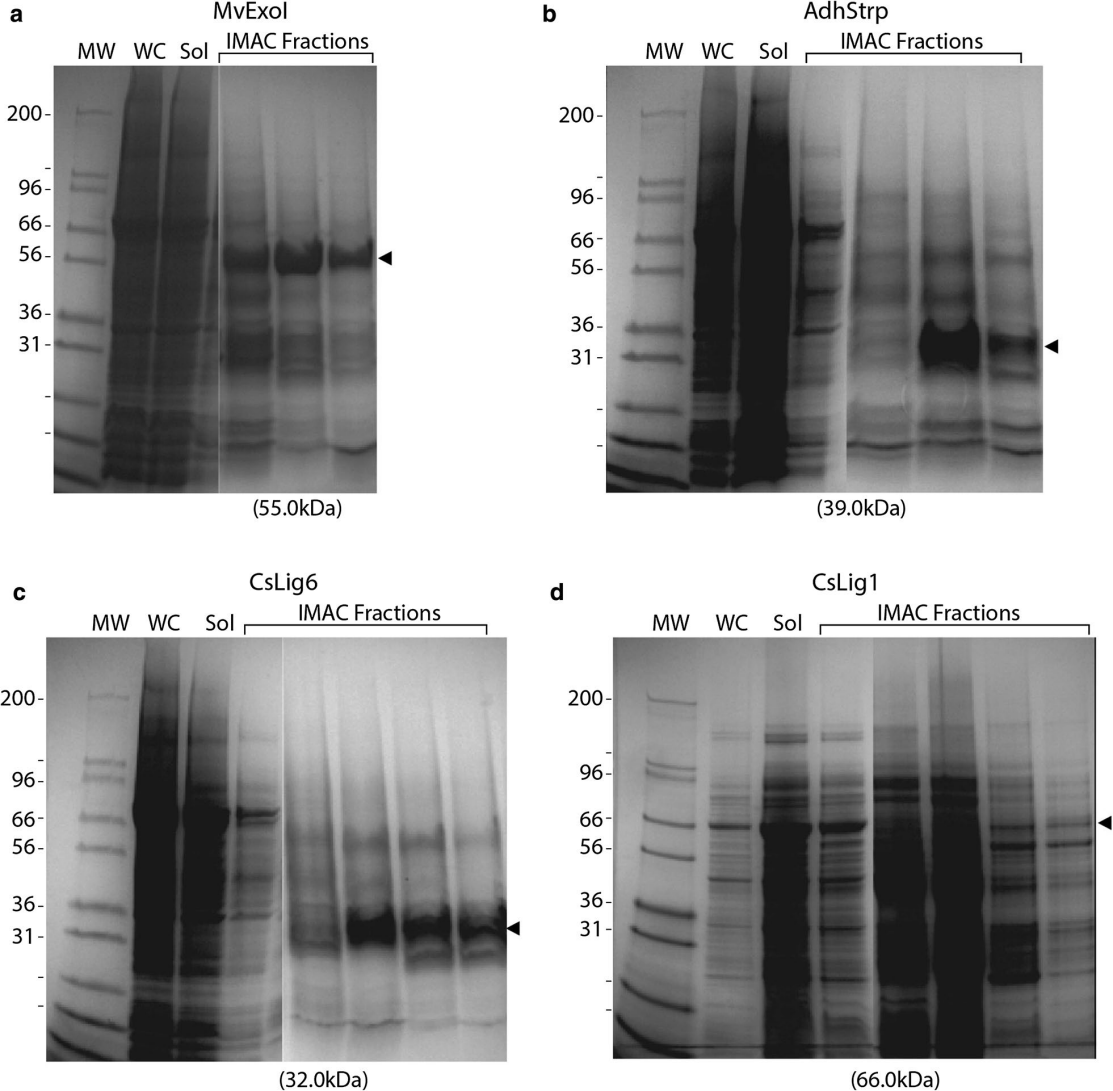
Expression and purification of non-*Aliivibrio* “test-case” enzymes. Enzymes from a wider phylogenetic range were selected as test-cases. **a** MvExoI (Mw—55 kDa), Exonucleae I from *Moritella viscosa.* **b** AdhStrep **(**Mw—39 kDa), alkoholdehydrogenase from *Streptomyces*. **c** CpLig6 (Mw—32 kDa), Ligase 6 from *Colwellia psychrerythraea*. **d** CsLig1 (Mw—66 kDa), Ligase 1 from *Cenarchaeum symbiosum.* Arrowheads indicate bands of expected size. MW = molecular weight marker. WC = whole cell extract, Sol = lysate soluble protein fraction. Immobilized metal affinity chromatography (IMAC) was done to purify 6 × His-tagged proteins, and proteins eluted in IMAC fractions are shown on the gels

These four genes were first cloned into the pTM214 vector, but downstream protein production experiments did not produce any detectable bands after SDS-PAGE. Therefore, the same enzyme genes were then cloned into pTM214 behind the 60-nt/20-aa fusion from gene AW0309160_00174 and conjugated into *A. wodanis* 03/09/160. Protein production was performed as described earlier. Figure 6 shows that bands corresponding to all four proteins are clearly visible on gels when samples of fractions collected after affinity purification of the His-tagged enzymes were run on SDS–polyacrylamide gels. Identity of bands was verified by mass spectrometry. Finally, activity of MvExoI was tested using the same assay as described for AsExoI (see above). The enzyme responds in a dose-dependent manner and is indeed expressed and purified in active form (Additional file 1: Fig. S3). This suggests that the 20-aa fusion does not interfere with enzyme activity.

To summarize, four enzymes originating from non-*Aliivibrio* organisms, including organisms very distantly related to *A. wodanis* (e.g. CsLig1 from Archaea) were expressed and purified. The activity of Exonuclease I from *M. viscosa* was tested and found to be active. Interestingly, the addition of a 60-nt/20-aa fusion, significantly increased the expression from not visible on gels to readily visible. Adapting to a stronger promoter system like T7 may increase expression/protein production. Thus far, the greatest benefit of the *A. wodanis* system is the apparent increase in successful protein folding of cold-adapted enzymes resulting from production of proteins at low temperature.

### A 25-aa peptide originating from AW0309160_00174 enhances export of sfGFP

**Fig. 7:**
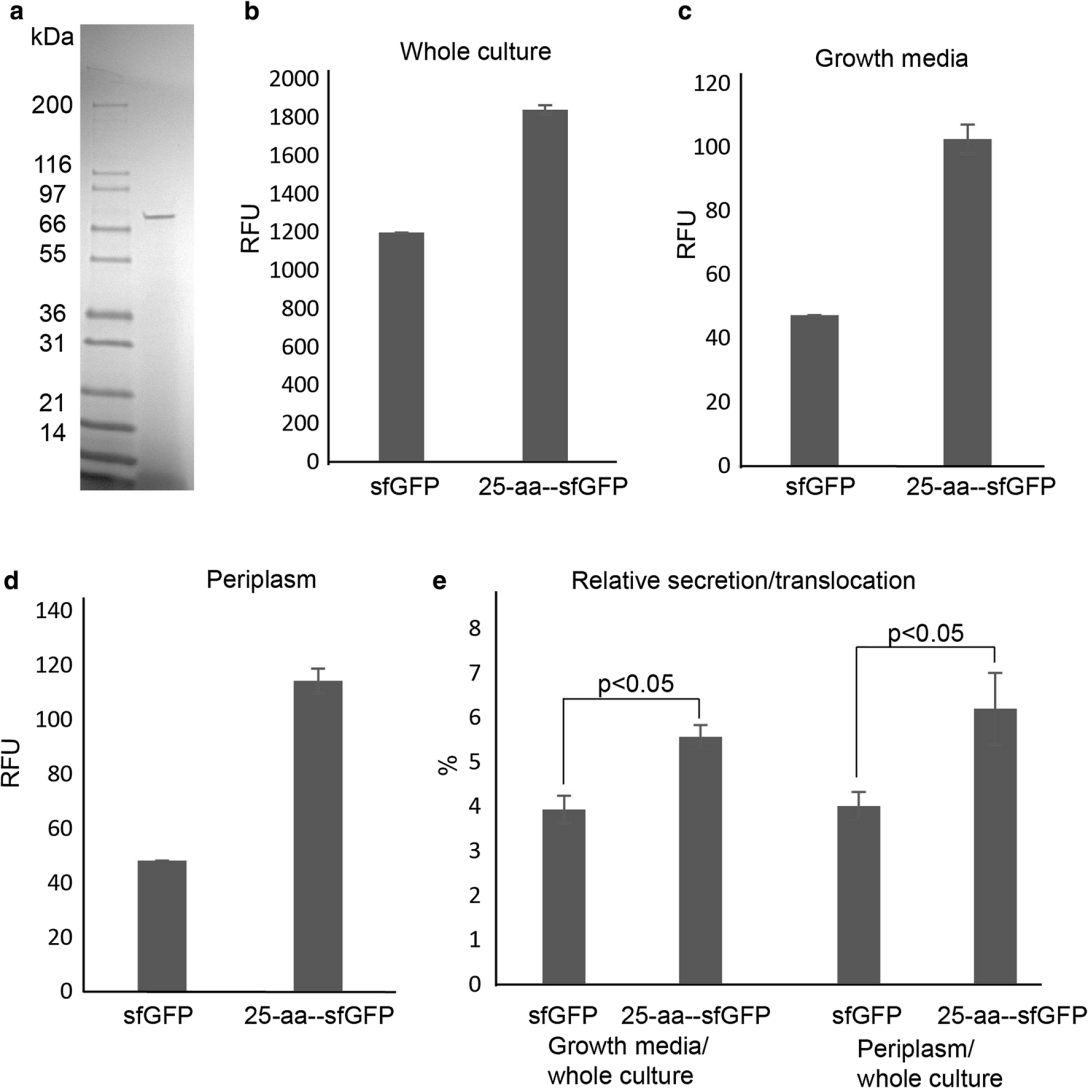
**a** SDS-PAGE of spent growth media of *A. wodanis* 03/09/160. The visible protein band was analyzed by LC–MS/MS and determined to originate from AW0309160_00174. **b–d** Measurement of fluorescence in whole culture, growth media and periplasm of *A. wodanis* 03/09/160 expressing sfGFP (from plasmid pTM214_sfGFP) or a 25-aa-sfGFP fusion (from plasmid pTM214_174ss_sfGFP). Bacteria without vector was used as control/blank). **e** Relative secretion/translocation compared to total florescence. p value calculated with Student’s t- test

Figure 7a shows an SDS-PAGE of 5× concentrated total proteins from spent media after 48 h of growth (to OD_600_ ~ 2) of *A. wodanis* 03/09/160 in a medium without high molecular weight proteins (5 g yeast extract, 25 g NaCl, 10 g casamino acids). A single band corresponding to a protein originating from AW0309160_00174 is readily visible [identified by Tandem mass spectrometry (MS–MS)]. This result shows that the highly expressed gene AW0309160_00174 is responsible for producing the corresponding protein, which is exported out of the cell. This is corroborated by SignalP [42] that predicts the presence of a 25-aa signal peptide in the N-terminus of the corresponding protein.

To test if the AW0309160_00174 signal sequence can be used to translocate recombinantly expressed proteins into the periplasm, or growth medium, a plasmid was constructed such that the 25-aa peptide was placed in front of the super folder GFP (sfGFP), resulting in a construct (named pTM214_174ss_sfGFP). When translocated to the periplasmic space, sfGFP is fluorescent [43]. A control construct (pTM214_sfGFP), which encodes sfGFP without the N-terminal 25-aa peptide was used in parallel experiments as a control, while *A. wodanis* 03/09/160 without vector was used as the control/blank. After 48 h of growth the fluorescence was determined in (i) growth media with cells, also referred to as “Whole Culture” (Fig. 7b), (ii) in the growth media (no cells) (Fig. 7c), and finally (iii) in the periplasm (Fig. 7d). Both sfGFP and the 25-aa-sfGFP fusion were detected in the growth media and periplasm. Interestingly, the 25-aa peptide significantly enhances the translocation/secretion of sfGFP (Fig. 7e). The relative fluorescence unit (RFU) measurements are approx. 2× when sfGFP is expressed as a 25-aa fusion. It has previously been reported that sfGFP itself can be used as a carrier protein in *E. coli* for secretion of recombinant fusion proteins, the authors of that study describe how the beta-barrel shape and negative charges on the molecule promotes translocation of the molecule [44]. This may explain the relatively high levels of secretion of sfGFP, even without the 25-aa signal peptide.

In summary, the first 25-aa originating from AW0309160_00174 enhance translocation of sfGFP into its surroundings, when used as an N-terminal fusion peptide. Secretion of recombinantly expressed proteins can have huge advantages, such as improved folding and post-translational modifications, easier downstream purification and processing, and compatibility with continuous culturing.

## Conclusion

In this work we have used the sub-Arctic bacterium *A. wodanis* as an expression host for “difficult-to-produce” enzymes. Basic characterization of 12 strains suggested that several strains were useful and that strain 03/09/160 was particularly suitable for expression. By using RNA-sequencing we revealed that a 60-nt/20-aa sequence of the most highly expressed gene can be used as a 5′-fusion to enhance expression of the downstream fusion partner. Three reporter systems and six enzymes were produced at low temperature, the activity of two of the enzymes was confirmed by molecular beacon-based assays. An N-terminal fusion of a 25-aa peptide and sfGFP showed that the peptide can be used as a signal for secretion of recombinantly produced proteins.

The current issue with our system is the lower level of produced proteins when compared to *E. coli.* To increase production of recombinant proteins in *A. wodanis* to levels similar to or higher than in *E. coli*, several different approaches are available. These include modification of strain to remove potentially damaging nucleases and proteinases as well as changing expression promoters to more efficient one. To increase recombinant protein production in non-modified strain, optimization of IPTG concentration would be first step, with increase of production time, use of autoinduction media and switching to high cell density culturing in bioreactors presenting other options.

The global market for specialty enzymes is continuously growing, driven by demands of the pharmaceutical industry, development of novel high-value enzymes, advancements in the biotechnology industry, the continued need for cost-efficient manufacturing process and calls for greener technologies. One major driver is an increasing demand for new enzymes that work efficiently at low temperatures, due to the growing demands for cleaner and less environmentally damaging technologies. This work contributes to the development of useful biotechnological tools to unlock further potential in developments in protein production systems, for the expression of cold-adapted enzymes, and possibly other unstable products such as, immunoglobulin fragments.

## Methods

### Bacterial strains and growth conditions

Twelve *A. wodanis* strains used in this study are listed in Table 1. Bacteria were revived from storage at − 80 °C by transferring frozen cells onto Blood agar or Marine agar plates, and placing them at 12 °C for 24–48 h. After being revived, the cells were grown in LB (Lysogeny Broth) supplemented with 2.5% NaCl liquid cultures for 1 week at 12 °C, or 2 weeks at 4 °C. Growth temperature was 12 °C with the exception of one experiment where expression of AsExoI was tested at 4 and 12 °C. In addition, during conjugative transfer of plasmids from *E. coli* CC118 λpir to *A. wodanis* where bacteria were grown in standard LB at 37 °C. Plasmid-carrying *A. wodanis* strains were always freshly made by conjugation before experiments and not revived from − 80 °C.

### Antibiotic resistance test

Antibiotic susceptibility testing was done by streaking *A. wodanis* cells onto LB plates supplemented with 2.5% NaCl and one of the following antibiotics: Chloramphenicol (2 µg/mL, Tetracycline (10 µg/mL), Carbenicillin (100 or 200 µg/mL) or Kanamycin (50 or 100 µg/mL). *A. wodanis* was regarded as susceptible (score = 0) if no growth was detected, as intermediately susceptible (score = 0.5) if poor growth was detected, or as resistant (score = 1) if good growth was detected. The concentrations of antibiotics tested were similar to the recommended working concentrations for *E. coli*, except Chloramphenicol which was tested at 2 µg/mL (instead of 25 µg/mL). If the test scored as “resistant”, then the test was repeated using 2× the recommended concentration. All *A. wodanis* strains were streaked onto the same agar plate and grown at 12 °C for 2 days.

### Conjugation and plasmid uptake test

The capacity of *A. wodanis* to receive conjugative plasmids was tested using a tri-parental mating approach. *E. coli* CC118 λpir (pEVS104) [45] as helper strain and *E. coli* CC118 λpir (pTM214) [36] was used as a donor. For genome integration *E. coli* CC118 λpir (pNQ705) was used. Integration was performed according to a previously described method [33, 46]. *E. coli* strains were grown to OD_600_ 0.5–0.7 in LB medium with kanamycin (50 µg/mL) and chloramphenicol (20 µg/mL), respectively, at 37 °C. “The recipient” *A. wodanis* was grown to OD_600_ = 1–2 in 3 mL LB supplemented with 2.5% NaCl at 12 °C. One mL of bacteria were next pelleted and resuspended in LB medium to original volume. After a second centrifugation and resuspension 500 µL of donor, helper and recipient bacteria were mixed and pelleted by centrifugation. The supernatant was removed and the pellet was resuspended in a small volume of residual LB medium of approximately 20 µL. The bacterial mix was spotted on an LB agar plate with 2.5% NaCl and incubated at 16 °C. Conjugates with replicating vectors, in this case pTM214, were incubated for 24 h, whereas those with integrating vectors, pNQ705, were incubated for 48 h. After incubation, bacteria were resuspended in LB with 2.5% NaCl and spread on selective agar containing 2 µg/mL chloramphenicol and incubated at 12 °C for 3 days. *E. coli* do not grow under these conditions. The number of colonies on agar plates were finally counted to assess the efficiency of DNA uptake. Plasmids were routinely transferred into *A. wodanis* as described above for pTM214 or pNQ705.

### Cloning

For test expression, genes of interest were PCR-amplified using primer pairs and a gateway plasmid (pET151/TEV/D-TOPO or pENTR/TEV/TOPO) containing the target gene as the template. Amplified DNA was inserted into the vector pTM214 using Fast cloning technique, according to protocol [47]. Primers and the resulting plasmids are shown in Additional file 2: Tables S1, S2. Additional file 2: Table S2 indicates that pTM214 contains an mCherry gene. When pTM214 was used as a vector for other genes the mCherry gene was not present as it was replaced by the new gene. Non-*Aliivibrio* test cases were expressed with or without a 5′ fusion partner (DNA sequence: 5′-ATGAGTAAGC AAATGAAGTT TGGACTTCTT CCAGCAGCGA TCGCTGGTGC ATTACTGAGC-3′) originating from *A. wodanis* 03/09/160 gene AW0309160_00174.

Superfolder GFP (sfGFP) [48, 49] was ordered as a synthetic construct including an N-terminal TEV-site for later fusion-proteins and C-terminal His-tag (GeneArt Strings from Thermo Fisher), and cloned into vector pTM214 using FastCloning with primers as stated in Additional file 2: Table S2. The resultant plasmid is named pTM214_sfGFP. To test if the AW0309160_00174 signal sequence can be used to translocate recombinantly expressed proteins into the periplasm or growth medium a plasmid was constructed such that the 25-aa peptide was placed in front of the super folder GFP (sfGFP) and cloned into pTM214 vector using FastCloning. The resulting construct was named pTM214_174ss_sfGFP.

### Recombinant production and purification of His-GFP

Six *A. wodanis* strains, conjugated with pTM214_His-GFP were grown in 15 mL cultures in LB with 2.5% NaCl and 2 µg/mL chloramphenicol and 0.1 mM IPTG at 12 °C shaking at 200 rpm. GFP was expressed for 3 days at 12 °C. 200 µL of culture was withdrawn from each sample and the cells pelleted in a micro centrifuge at 13 K rpm for 5 min. The bacterial pellet was lysed with 30 µL BugBuster (MerckMillipore) according to the manufacturer’s protocol, incubated at room temperature for 30 min and finally pelleted. 25 µL of each supernatant was used to measure GFP fluorescence in a Spectramax Gemini (Molecular Devices) spectrophotometer at wavelength 485–538 nm.

### Recombinant production and purification of test cases

Protein production was done in strains K7F1 150913, 02/09/382, 01/09/401, 88/09/441, 04/60/17347 and 03/09/160 to verify that strains producing the highest amounts of RFP (Fig. 1c) also perform best when producing cold-adapted enzymes. Protein production was done in 15 mL LB supplemented with 2.5% NaCl, 2 µg/mL chloramphenicol and 0.1 mM IPTG for 3 days at 12 °C. After 3 days of expression, samples of whole cell extracts and soluble proteins were separated by SDS-PAGE. The same strains were used to produce AsExoI to verify protein production under the same conditions. The proteins produced were purified using His-tagged magnetic beads (His Mag Sepharose Ni, GE healthcare) according to protocol and separated using SDS page.

For large scale production and purification test-case proteins were produced in *A. wodanis* 03/09/160 from their respective plasmids (see Additional file 2: Table S2) by growing the bacterium in 1 L LB supplemented with 2.5% NaCl, 2 µg/mL chloramphenicol and 0.1 mM IPTG for 3 days at 12 °C. Cells were then spun down (6000 rpm, 30 min, 12 °C) and lysed in 30 mL lysis buffer (50 mM Tris pH 8.0, 750 mM NaCl, and 5% (v/v) glycerol) supplemented with 1× Complete protease inhibitor cocktail (Roche) and 1 U/µL HL/SAN DNase (ArcticZymes). The cells were disrupted using a cell disruptor (Constant Systems, Ltd.) at 1.38 kbar for four cycles. The lysate was cleared by centrifugation at 20,000×*g* for 30 min at 4 °C. Affinity purification of test proteins was carried out on a 5 mL HisTrap HP column (GE Healthcare) equilibrated with buffer A (50 mM Tris pH 8.0, 750 mM NaCl, 5% (v/v) glycerol and 10 mM imidazole) using an *Ä*KTA purifier (GE Healthcare). The bound protein was eluted across a gradient of 0–100% buffer B (50 mM Tris pH 8.0, 750 mM NaCl, 5% (v/v) glycerol and 500 mM imidazole). The purity of the protein was evaluated by SDS-PAGE and the identity of proteins was verified using a Tandem mass spectrometry (MS–MS) service at the Tromsø University Proteomics Platform (TUPP), using Orbitrap Fusion Lumos or a Q-Exactive HF-X and analyzing data across NCBI bacteria and all entries and Swissprot, all entries databases.

### Enzyme activity assays

The two enzyme activity assays used in this work are both based on so-called “molecular beacons”. Each “molecular beacon” consists of a hairpin shaped DNA oligonucleotide with an internally quenched fluorophore (in this case FAM). TAMRA was used as the FAM quencher. Enzyme activities of affinity purified AsExoI and MvExoI were tested in 50 µL reactions containing the following: 0.2 µM ssDNA “molecular beacon” substrate (5′-FAM-CGCCATCGGAGGTTC-TAMRA-3′), 50 mM Tris pH 8.5, 30 mM MgCl_2_, 1 mM DTT, 0.2 mg/mL BSA, 2% glycerol and 8.5 nM enzyme (Exonuclease I). The reaction was carried out in a black 96-well fluorescence assay plate (Corning^®^) and the increase in FAM fluorescence (excitation at 485 nm, emission at 518 nm) was measured as relative fluorescence units (RFU) at appropriate time intervals for over 40 min.

The activity assay for AwPolII was based on a molecular beacon probe (5′-GGCCCGTDabcylAGGAGGAAAGGACATCTTCTAGCATFAMACGGGCCGTCAAGTTCATG GCCAGTCAAGTCGTCAGAAATTTCGCACCAC-3′) (modified from [50]). The molecular beacon template consists of a 23mer loop that is connected by a GC-rich 8mer stem region. The 8-mer stem region consists of two 8 nucleotide sequences (indicated in italics) and a 43mer extension. The fluorophores Dabcyl and FAM are attached to the indicated “T” nucleotides. Due to the loop formation the fluorophores Dabcyl and FAM are in close proximity and thus quenched. Upon extension by DNA polymerase I of the primer (5′-GTGGTGCGAAATTTCTGAC-3′) that is annealed to the molecular beacon template the stem is opened and the increase in distance of the two fluorophores is measured by the restoration of FAM fluorescence (excitation 485 nm, emission 518 nm). The assay was monitored in 50 µL reactions containing 0.2 µM substrate (molecular beacon) mixed with 0.2 mM dNTP in 1× reaction buffer (250 mM Tris–HCl, pH 8.5, 250 mM, KCl, 25 mM MgCl2) and 1× DB (1 mg/mL BSA, 5 mM DTT, 10% glycerol). The mixture was first incubated at 25 °C for 5 min, and the reaction was started by adding the purified enzyme. Increase in fluorescence was measured (excitation 485 nm, emission 518 nm) for 15 min at 10 s intervals (total of 91 reads). All measurements were performed in Corning black 96-well plate (Sigma Aldrich, CLS3991-25EA).

### Isolation of proteins in growth media for SDS-PAGE

*A. wodanis* 03/09/160 was grown in a media with 5 g yeast extract, 25 g NaCl and 10 g casamino acids. This media does not contain any high molecular weight protein when analysed on SDS-PAGE. The strain was grown for 48 h reaching OD_600_ = 2. The culture was spun down and the supernatant was sterile filtered through a 0.45 µM filter. The spent growth medium was then up-concentrated 5 times using a spin filter with 3 K cut-off, to a protein concentration of 6.3 mg/mL. The concentrated growth medium was analyzed on SDS-PAGE and the protein was identified by the Tandem mass spectrometry (MS–MS) service at the Tromsø University Proteomics Platform (TUPP).

### Isolation of periplasmic proteins

A culture of *A. wodanis* was centrifuged and the pellet was resuspended in a volume corresponding to 1/10 of the original volume in periplasmic lysis buffer (0.2 M Tris–HCl pH 8.0, 200 g/L sucrose and 0.1 M EDTA). The suspension was incubated on ice for 20 min followed by centrifugation. A second step with MgCl_2_ resulted in complete lysis of the cells. Here cold 5 mM MgCl_2_ was added to the initial culture (1/20th the volume) and mixed. It was incubated for 20 min on ice after which whole mixture was centrifuge at 5000*g* for 40 min at 4 °C. Supernatant containing periplasmic proteins was collected and used in further analysis.

### Quantification of sfGFP in growth media and periplasm

*A. wodanis* 03/09/160 with or without vector containing sfGFP was grown in standard conditions in the presence of IPTG for 48 h. The fluorescence was determined using a Specktramax photometer (ex 485 nm, em 525 nm) in the following samples: 100 µL whole culture (media with cells), 100 µL supernatant and 50 µL of the periplasmic fraction (prepared as above).

### RNA sequencing, genome sequencing and bioinformatics analyses

To find the most highly expressed genes *A. wodanis* 03/09/160 was first grown under standard growth conditions: LB supplemented with 2.5% NaCland harvested at OD_600nm_ = 2 (exponential phase). After cultivation, total RNA was purified from cell pellets using the Masterpure complete DNA and RNA purification kit (Epicentre) following the manufacturer’s protocol. The RNA quality was then determined using a Bioanalyzer and a Prokaryote Total RNA Pico Chip (Agilent Technologies). Five µg total RNA was then used in the Ribo-Zero rRNA Removal Kit (bacteria) (Epicentre) according to manufacturer instructions to remove ribosomal (r) RNA. Samples depleted in rRNA were ethanol precipitated and analyzed on a Bioanalyzer using mRNA Pico Chips (Agilent Technologies). RNA-sequencing libraries were generated from rRNA-depleted RNA samples using the ScriptSeq Complete library prep kit (Illumina) in combination with library size selection using a Pippin Prep cassette (Sage Science). The size selected cDNA library was sequenced with MiSeq Reagent Kit v3 with 2 × 75 bp read length over 150 cycles, generating 25 mill reads and 3.75 Gb.

Reads were quality checked using FastQC. Further analysis of the RNA-Seq data was performed using a Galaxy pipeline consisting of EDGE-pro v1.0.1 (Estimated Degree of Gene Expression in Prokaryotes) and DESeq to align the reads to the *A. wodanis* 03/09/160 genome, and estimate gene expression value as “baseMean” (mean expression level across all replicates). The RNA seq datasets are publically available at GEO (NCBI) or ArrayExpress (EBI) using the accession number PRJEB30658..

### Genome sequencing

Total DNA was isolated from *A. wodanis* 03/09/160 grown under standard conditions to stationary phase using Genomic-tip 100/g (Qiagen) according to the manufacturer’s protocol. The final DNA concentration and quality were measured using a Nanodrop 2000c (Thermo Scientific) instrument, and the integrity of high molecular weight DNA was examined on a 1% agarose gel. Genomic DNA was sequenced at the Norwegian Sequencing Centre (NSC) using the PacBio technology platform. Libraries were constructed using the PacBio 20 kb library preparation protocol. Size selection of the final library was performed using BluePippin with a 7 kb cut-off. Libraries were sequenced on a Pacific Biosciences RS II instrument using P6-C4 chemistry with 360 min movie time. PacBio reads were assembled using HGAP v3 [51], and Minimus2 [52] was used to circularize contigs. RS_Resequencing.1 software (SMRT Analysis version v2.3.0) was used to map reads back to assembled and circularized sequence in order to correct sequence after circularization.

A final refinement of the genome sequence derived from the PacBio instrument was done by re-sequencing the *A. wodanis* 03/09/160 genome using a MiSeq Illumina instrument and paired-end sequencing (i.e., 2 × 300-bp reads). The resulting reads were mapped onto the PacBio derived circularized sequence using the Bowtie 2 software to correct for potential single nucleotide errors that may occur due to the high error rate during PacBio sequencing [53]. The final genome sequence is publically available at ENA/GenBank/DDBJ using the accession number PRJEB30658.

## Supplementary information

**Additional file 1.** Additional figures S1–S3.

**Additional file 2.** Additional tables S1–S2.

## Data Availability

All data generated or analyzed in this study are included in this manuscript or in Additional files, or can be accessed from the publically available European Nucleotide Archive.
