# Supplementary material for: Aliivibrio wodanis as a production host: development of genetic tools for expression of cold-active enzymes
Source: Microb Cell Fact. 2019 Nov 11;18:197. doi: 10.1186/s12934-019-1247-1 (PMC6844050; doi:10.1186/s12934-019-1247-1)
Supplement: Supplementary file 1 — Additional file 1. Additional figures S1–S3. [file 12934_2019_1247_MOESM1_ESM.pptx]

## Slide 1
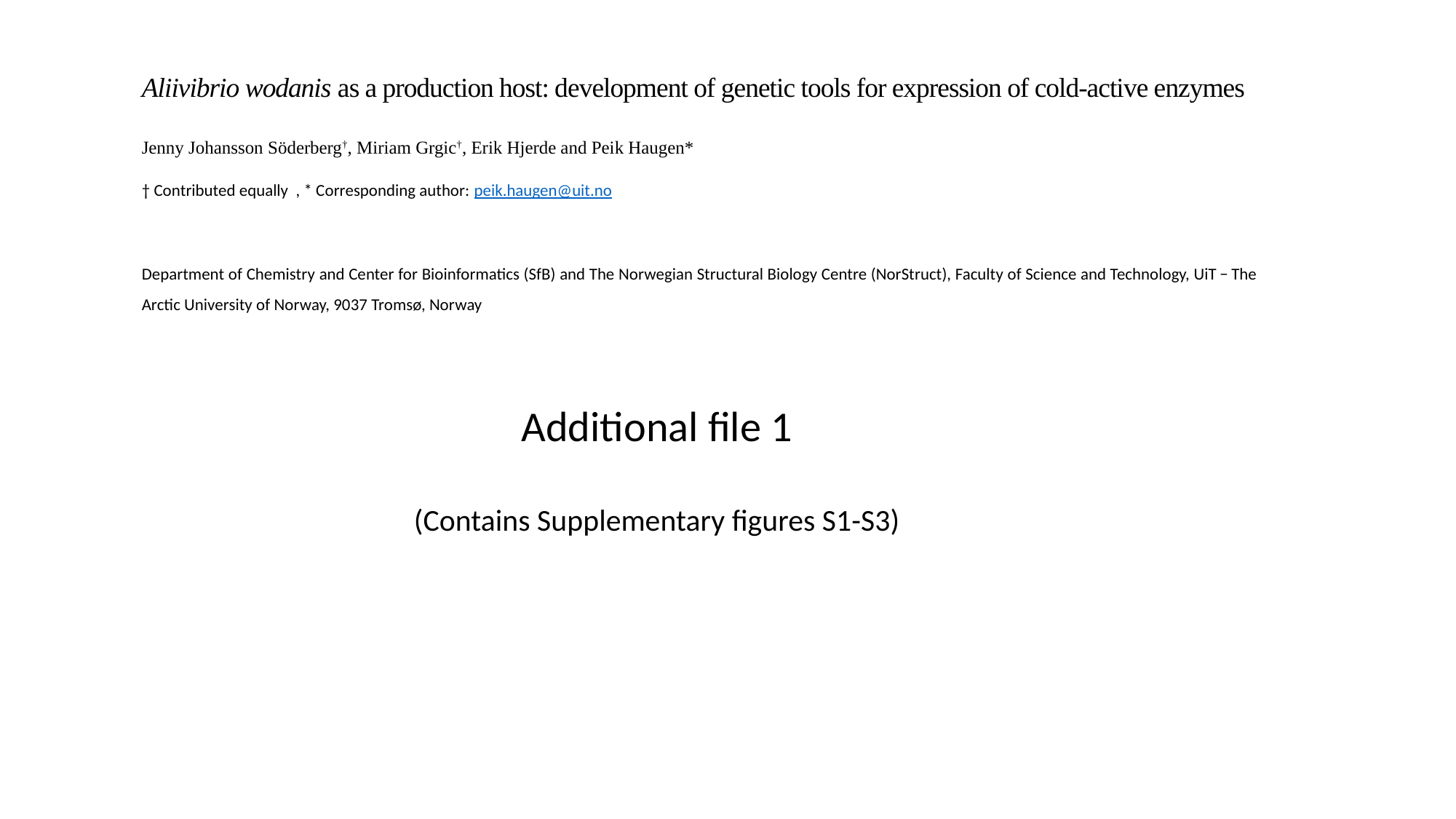

Aliivibrio wodanis as a production host: development of genetic tools for expression of cold-active enzymes
Jenny Johansson Söderberg†, Miriam Grgic†, Erik Hjerde and Peik Haugen*
† Contributed equally , * Corresponding author: peik.haugen@uit.no
Department of Chemistry and Center for Bioinformatics (SfB) and The Norwegian Structural Biology Centre (NorStruct), Faculty of Science and Technology, UiT − The Arctic University of Norway, 9037 Tromsø, Norway
Additional file 1
(Contains Supplementary figures S1-S3)

## Slide 2
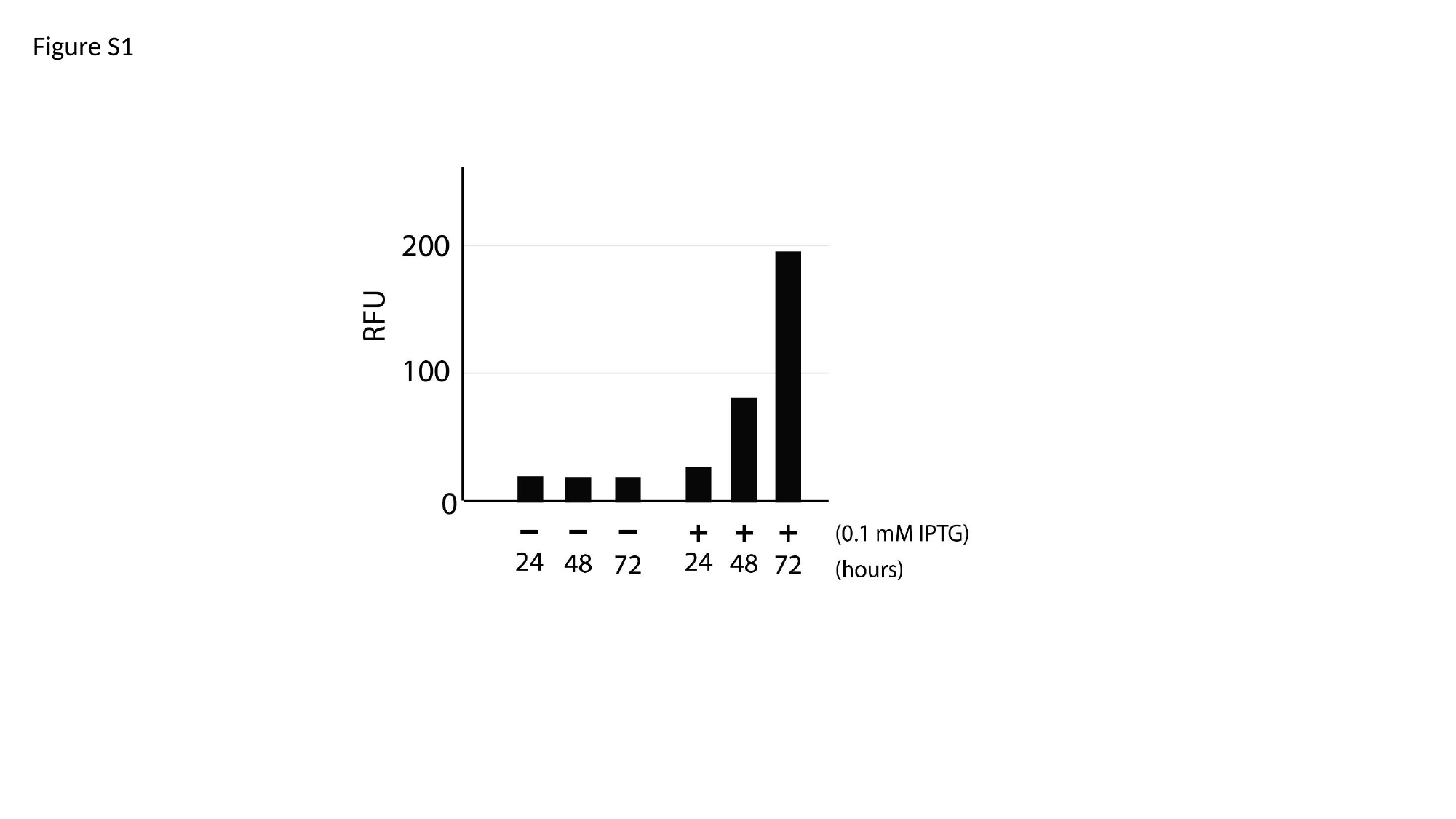

Figure S1

## Slide 3
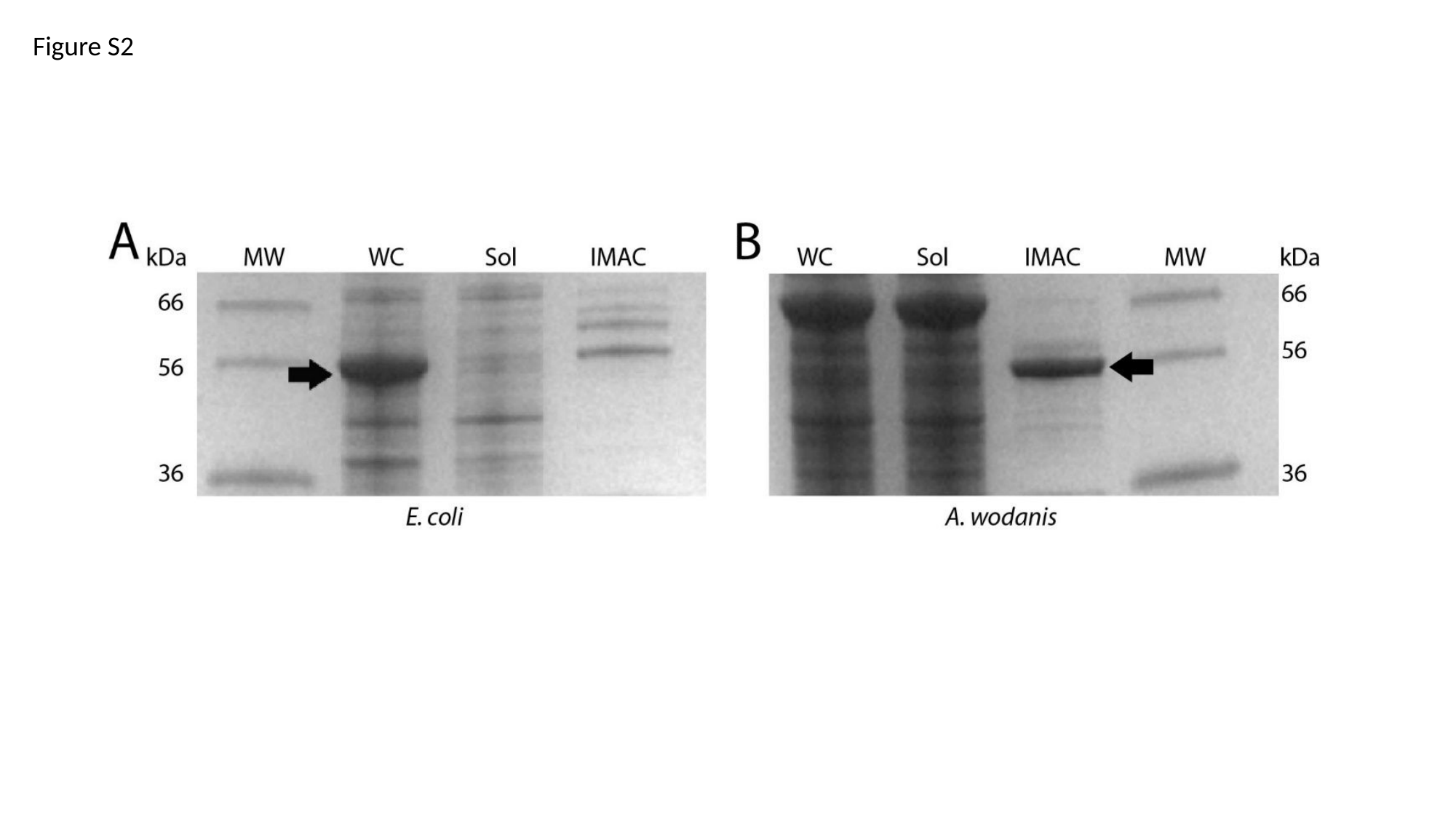

Figure S2

## Slide 4
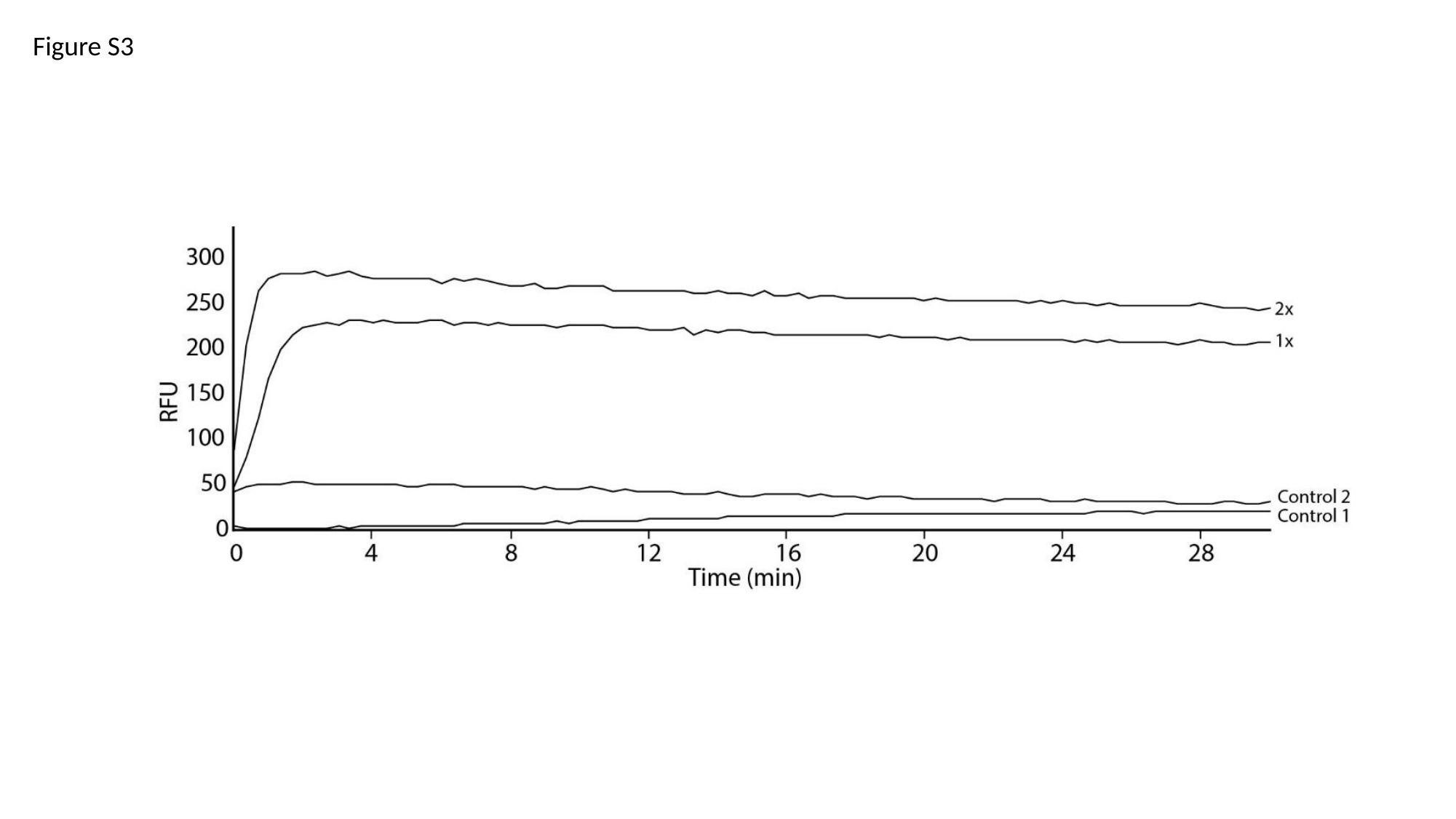

Figure S3
