# Supplementary material for: Aliivibrio wodanis as a production host: development of genetic tools for expression of cold-active enzymes
Source: Microb Cell Fact. 2019 Nov 11;18:197. doi: 10.1186/s12934-019-1247-1 (PMC6844050; doi:10.1186/s12934-019-1247-1)
Supplement: Supplementary file 2 — Additional file 2. Additional tables S1–S2. [file 12934_2019_1247_MOESM2_ESM.pptx]

## Slide 1
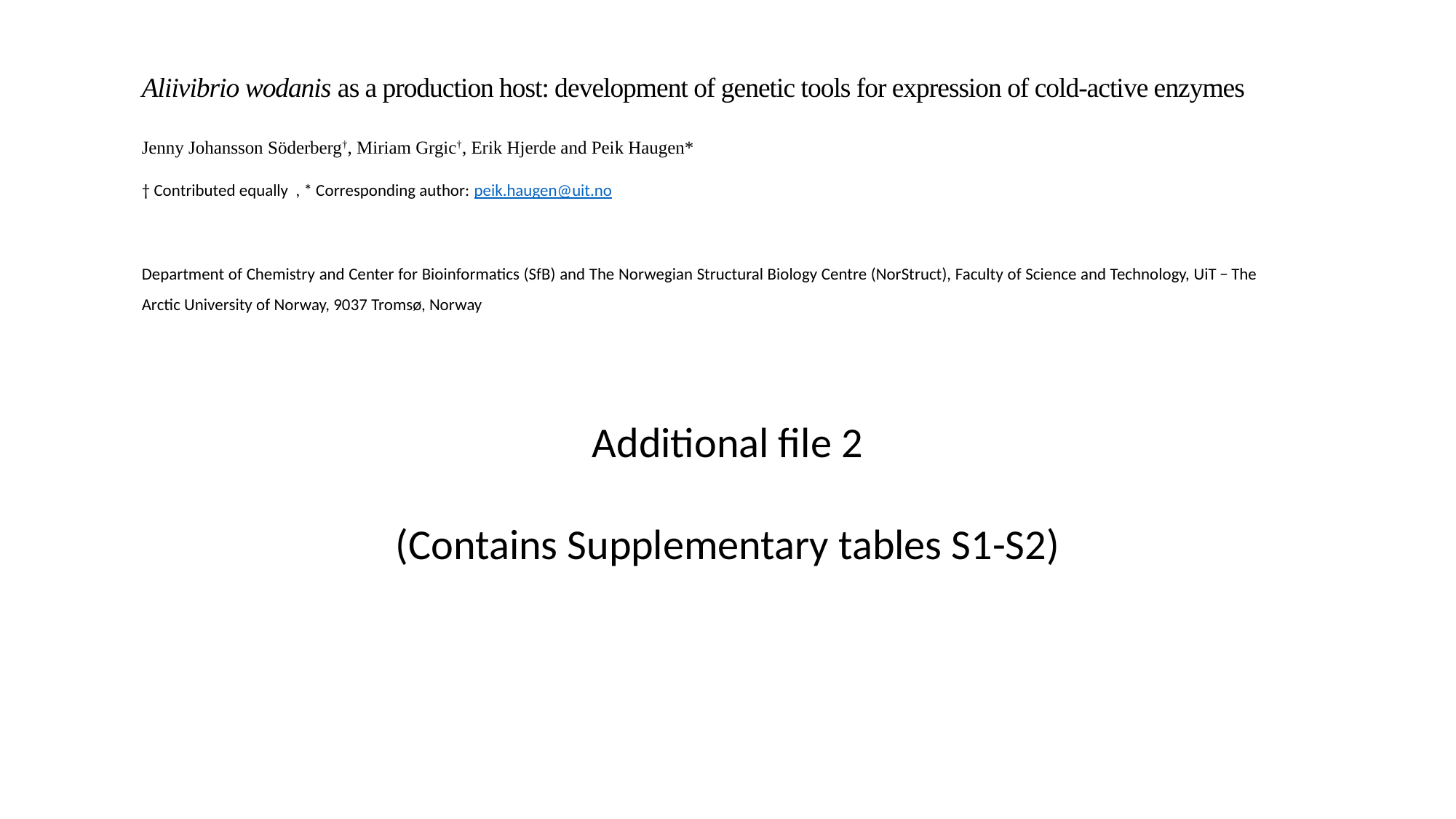

Aliivibrio wodanis as a production host: development of genetic tools for expression of cold-active enzymes
Jenny Johansson Söderberg†, Miriam Grgic†, Erik Hjerde and Peik Haugen*
† Contributed equally , * Corresponding author: peik.haugen@uit.no
Department of Chemistry and Center for Bioinformatics (SfB) and The Norwegian Structural Biology Centre (NorStruct), Faculty of Science and Technology, UiT − The Arctic University of Norway, 9037 Tromsø, Norway
Additional file 2
(Contains Supplementary tables S1-S2)

## Slide 2
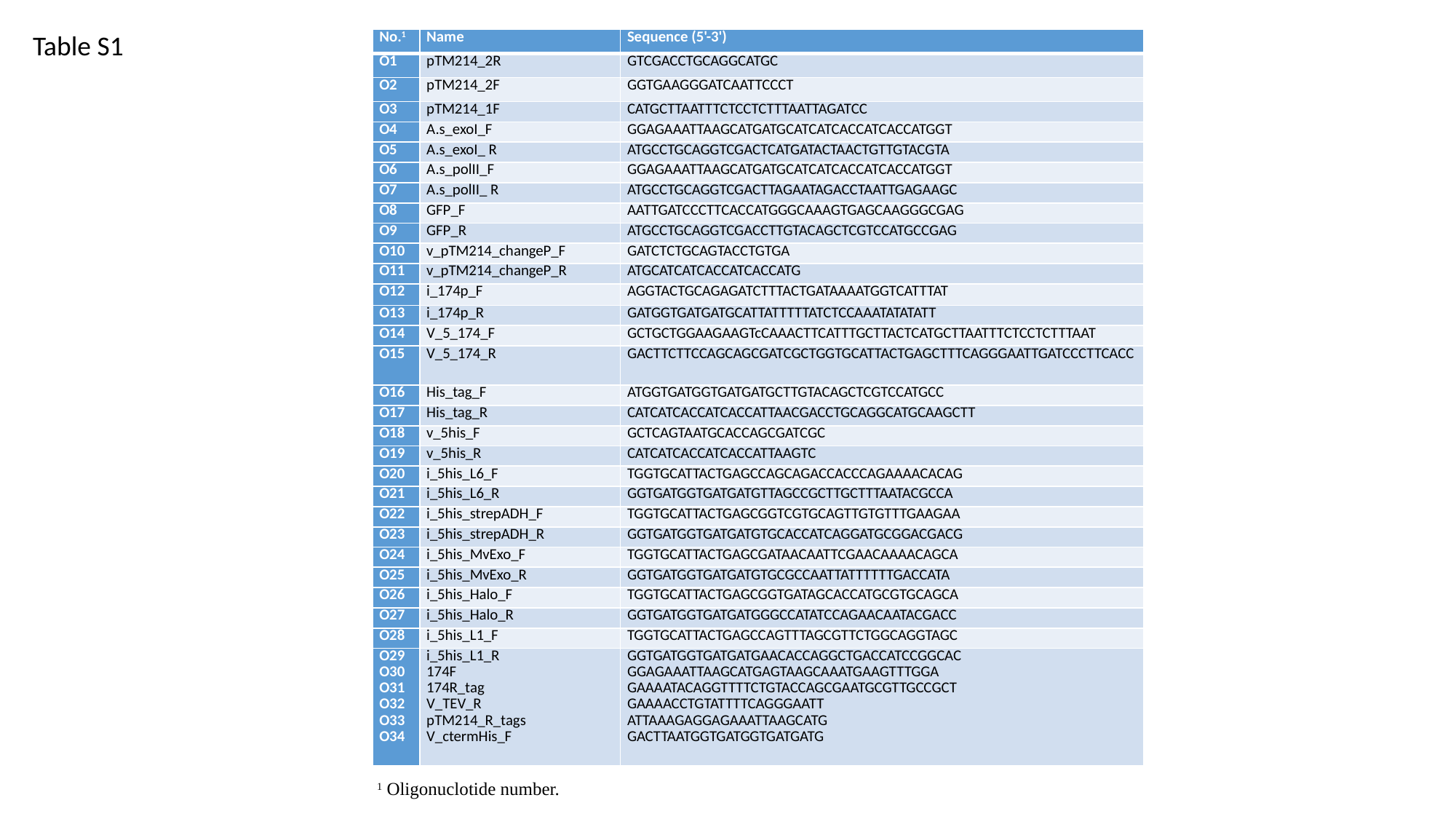

Table S1
| No.1 | Name | Sequence (5'-3') |
| --- | --- | --- |
| O1 | pTM214\_2R | GTCGACCTGCAGGCATGC |
| O2 | pTM214\_2F | GGTGAAGGGATCAATTCCCT |
| O3 | pTM214\_1F | CATGCTTAATTTCTCCTCTTTAATTAGATCC |
| O4 | A.s\_exoI\_F | GGAGAAATTAAGCATGATGCATCATCACCATCACCATGGT |
| O5 | A.s\_exoI\_ R | ATGCCTGCAGGTCGACTCATGATACTAACTGTTGTACGTA |
| O6 | A.s\_polII\_F | GGAGAAATTAAGCATGATGCATCATCACCATCACCATGGT |
| O7 | A.s\_polII\_ R | ATGCCTGCAGGTCGACTTAGAATAGACCTAATTGAGAAGC |
| O8 | GFP\_F | AATTGATCCCTTCACCATGGGCAAAGTGAGCAAGGGCGAG |
| O9 | GFP\_R | ATGCCTGCAGGTCGACCTTGTACAGCTCGTCCATGCCGAG |
| O10 | v\_pTM214\_changeP\_F | GATCTCTGCAGTACCTGTGA |
| O11 | v\_pTM214\_changeP\_R | ATGCATCATCACCATCACCATG |
| O12 | i\_174p\_F | AGGTACTGCAGAGATCTTTACTGATAAAATGGTCATTTAT |
| O13 | i\_174p\_R | GATGGTGATGATGCATTATTTTTATCTCCAAATATATATT |
| O14 | V\_5\_174\_F | GCTGCTGGAAGAAGTcCAAACTTCATTTGCTTACTCATGCTTAATTTCTCCTCTTTAAT |
| O15 | V\_5\_174\_R | GACTTCTTCCAGCAGCGATCGCTGGTGCATTACTGAGCTTTCAGGGAATTGATCCCTTCACC |
| O16 | His\_tag\_F | ATGGTGATGGTGATGATGCTTGTACAGCTCGTCCATGCC |
| O17 | His\_tag\_R | CATCATCACCATCACCATTAACGACCTGCAGGCATGCAAGCTT |
| O18 | v\_5his\_F | GCTCAGTAATGCACCAGCGATCGC |
| O19 | v\_5his\_R | CATCATCACCATCACCATTAAGTC |
| O20 | i\_5his\_L6\_F | TGGTGCATTACTGAGCCAGCAGACCACCCAGAAAACACAG |
| O21 | i\_5his\_L6\_R | GGTGATGGTGATGATGTTAGCCGCTTGCTTTAATACGCCA |
| O22 | i\_5his\_strepADH\_F | TGGTGCATTACTGAGCGGTCGTGCAGTTGTGTTTGAAGAA |
| O23 | i\_5his\_strepADH\_R | GGTGATGGTGATGATGTGCACCATCAGGATGCGGACGACG |
| O24 | i\_5his\_MvExo\_F | TGGTGCATTACTGAGCGATAACAATTCGAACAAAACAGCA |
| O25 | i\_5his\_MvExo\_R | GGTGATGGTGATGATGTGCGCCAATTATTTTTTGACCATA |
| O26 | i\_5his\_Halo\_F | TGGTGCATTACTGAGCGGTGATAGCACCATGCGTGCAGCA |
| O27 | i\_5his\_Halo\_R | GGTGATGGTGATGATGGGCCATATCCAGAACAATACGACC |
| O28 | i\_5his\_L1\_F | TGGTGCATTACTGAGCCAGTTTAGCGTTCTGGCAGGTAGC |
| O29 O30 O31 O32 O33 O34 | i\_5his\_L1\_R 174F 174R\_tag V\_TEV\_R pTM214\_R\_tags V\_ctermHis\_F | GGTGATGGTGATGATGAACACCAGGCTGACCATCCGGCAC GGAGAAATTAAGCATGAGTAAGCAAATGAAGTTTGGA GAAAATACAGGTTTTCTGTACCAGCGAATGCGTTGCCGCT GAAAACCTGTATTTTCAGGGAATT ATTAAAGAGGAGAAATTAAGCATG GACTTAATGGTGATGGTGATGATG |
1 Oligonuclotide number.

## Slide 3
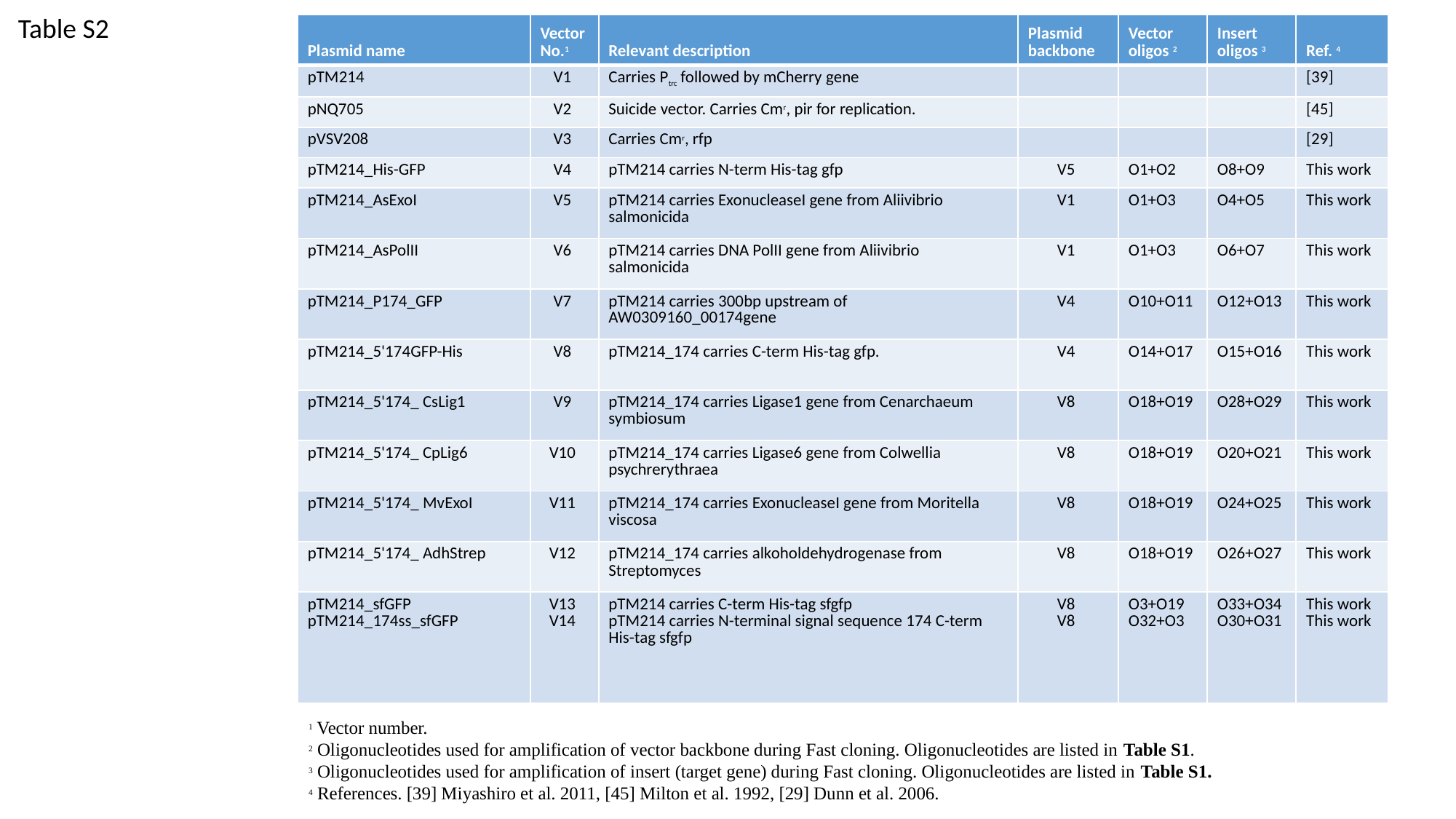

Table S2
| Plasmid name | Vector No.1 | Relevant description | Plasmid backbone | Vector oligos 2 | Insert oligos 3 | Ref. 4 |
| --- | --- | --- | --- | --- | --- | --- |
| pTM214 | V1 | Carries Ptrc followed by mCherry gene | | | | [39] |
| pNQ705 | V2 | Suicide vector. Carries Cmr, pir for replication. | | | | [45] |
| pVSV208 | V3 | Carries Cmr, rfp | | | | [29] |
| pTM214\_His-GFP | V4 | pTM214 carries N-term His-tag gfp | V5 | O1+O2 | O8+O9 | This work |
| pTM214\_AsExoI | V5 | pTM214 carries ExonucleaseI gene from Aliivibrio salmonicida | V1 | O1+O3 | O4+O5 | This work |
| pTM214\_AsPolII | V6 | pTM214 carries DNA PolII gene from Aliivibrio salmonicida | V1 | O1+O3 | O6+O7 | This work |
| pTM214\_P174\_GFP | V7 | pTM214 carries 300bp upstream of AW0309160\_00174gene | V4 | O10+O11 | O12+O13 | This work |
| pTM214\_5'174GFP-His | V8 | pTM214\_174 carries C-term His-tag gfp. | V4 | O14+O17 | O15+O16 | This work |
| pTM214\_5'174\_ CsLig1 | V9 | pTM214\_174 carries Ligase1 gene from Cenarchaeum symbiosum | V8 | O18+O19 | O28+O29 | This work |
| pTM214\_5'174\_ CpLig6 | V10 | pTM214\_174 carries Ligase6 gene from Colwellia psychrerythraea | V8 | O18+O19 | O20+O21 | This work |
| pTM214\_5'174\_ MvExoI | V11 | pTM214\_174 carries ExonucleaseI gene from Moritella viscosa | V8 | O18+O19 | O24+O25 | This work |
| pTM214\_5'174\_ AdhStrep | V12 | pTM214\_174 carries alkoholdehydrogenase from Streptomyces | V8 | O18+O19 | O26+O27 | This work |
| pTM214\_sfGFP  pTM214\_174ss\_sfGFP | V13  V14 | pTM214 carries C-term His-tag sfgfp  pTM214 carries N-terminal signal sequence 174 C-term His-tag sfgfp | V8  V8 | O3+O19  O32+O3 | O33+O34  O30+O31 | This work  This work |
1 Vector number.
2 Oligonucleotides used for amplification of vector backbone during Fast cloning. Oligonucleotides are listed in Table S1.
3 Oligonucleotides used for amplification of insert (target gene) during Fast cloning. Oligonucleotides are listed in Table S1.
4 References. [39] Miyashiro et al. 2011, [45] Milton et al. 1992, [29] Dunn et al. 2006.
